# A case report and literature review on new-onset systemic lupus erythematosus leading to thrombocytopenia in a hemodialysis patient

**DOI:** 10.1097/MD.0000000000042820

**Published:** 2025-06-13

**Authors:** Zhe Zhang, Yunshi Lai, Xiaoyi Liu, Peiyi Ye, Yaozhong Kong, Chao Xie

**Affiliations:** a Department of Nephrology, The First People’s Hospital of Foshan, Foshan, Guangdong, China.

**Keywords:** case report, hemodialysis, systemic lupus erythematosus, thrombocytopenia

## Abstract

**Rationale:**

Systemic lupus erythematosus (SLE) is a complex autoimmune disease that affects various organs. Disease activity in SLE may diminish following the initiation of dialysis in patients with end-stage renal disease.

**Patient concern:**

We report the case of a 69-year-old female patient with a prior diagnosis of IgA nephropathy who developed SLE characterized by severe thrombocytopenia after hemodialysis.

**Diagnoses:**

The patient presented with fever, rash, polyarthralgia, thrombocytopenia, hemolytic anemia, positive antinuclear antibodies, anti-nucleosome antibodies, anticardiolipin antibodies, anti-β2-glycoprotein I antibodies, and decreased complement C3 and C4 levels. She was diagnosed with SLE complicated by hematological damage and immune thrombocytopenia.

**Interventions:**

The treatment included an intravenous infusion of 5% human immunoglobulin at 20 g/day for 5 days combined with an intravenous infusion of methylprednisolone at 500 mg/day for 3 days. Plasma exchange therapy was conducted a total of 3 times.

**Outcomes:**

The patient was discharged with methylprednisolone and hydroxychloroquine treatment. The platelet count was stable, antinuclear antibody, anti-nucleosome antibody, antiphospholipid antibody, Coombs test, and complement C3 and C4 levels were normal after discharge.

**Lessons:**

In patients with unexplained thrombocytopenia, the possibility of SLE should be considered even after hemodialysis initiation.

## 1. Introduction

Systemic lupus erythematosus (SLE) is an autoimmune disease that primarily affects multiple organs, including the skin, joints, central nervous system, and kidneys.^[1]^ SLE typically affects young and middle-aged women, with few cases occurring after menopause.^[2]^ Previous studies indicated that disease activity in SLE often decreases after starting dialysis treatment for end-stage renal disease.^[3,4]^ Consequently, only a few cases of new-onset SLE during maintenance dialysis have been reported. Here, we present a rare case of a 69-year-old woman with IgA nephropathy who developed SLE resulting in severe thrombocytopenia after undergoing hemodialysis.

## 2. Case presentation

**Figure 1.**
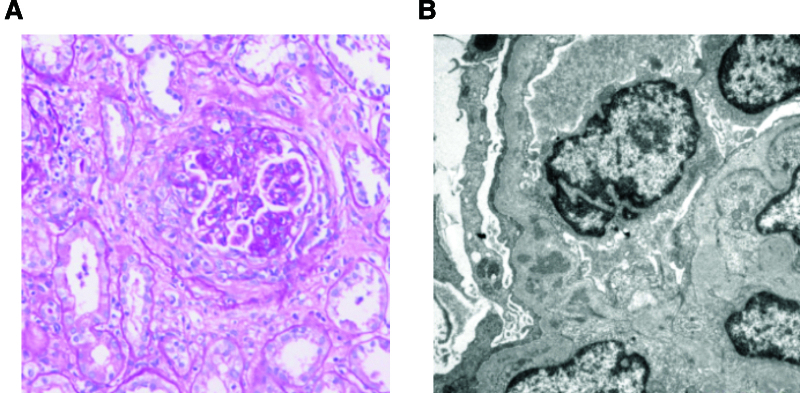
The renal pathological results of the patient. (A) HE staining results showing moderate mesangial proliferative IgA nephropathy with focal sclerosis. (B) Electron microscopy results confirming the diagnosis of IgA nephropathy.

A 69-year-old female patient presented with increased frequency of nocturnal urination in 2015. Her serum creatinine level was 249 μmol/L, urine protein was 0.92 g/24 h, and renal biopsy revealed moderate mesangial proliferative glomerulonephritis. During outpatient follow-up, her serum creatinine level gradually increased. A repeat renal biopsy in 2019 showed moderate mesangial proliferative IgA nephropathy with focal sclerosis (Fig. 1), and the patient received symptomatic treatments, including antihypertensive therapies. In June 2023, her creatinine level increased to 976 μmol/L, which was accompanied by fatigue, poor appetite, and pruritus. On June 12, 2023, a right forearm arteriovenous fistula was created to prepare for dialysis.

The patient developed edema in both lower limbs along with fatigue, poor appetite, and pruritus. Her urine output was significantly reduced and she was admitted to the hospital on July 13, 2023. Physical examination revealed a temperature of 37.2℃, pulse of 75 beats/min, and blood pressure of 121/59 mm Hg. Scattered dark erythematous patches, papules, scratches, and desquamation were observed throughout her body, along with dense pinprick petechiae in both lower limbs.

**Figure 2.**
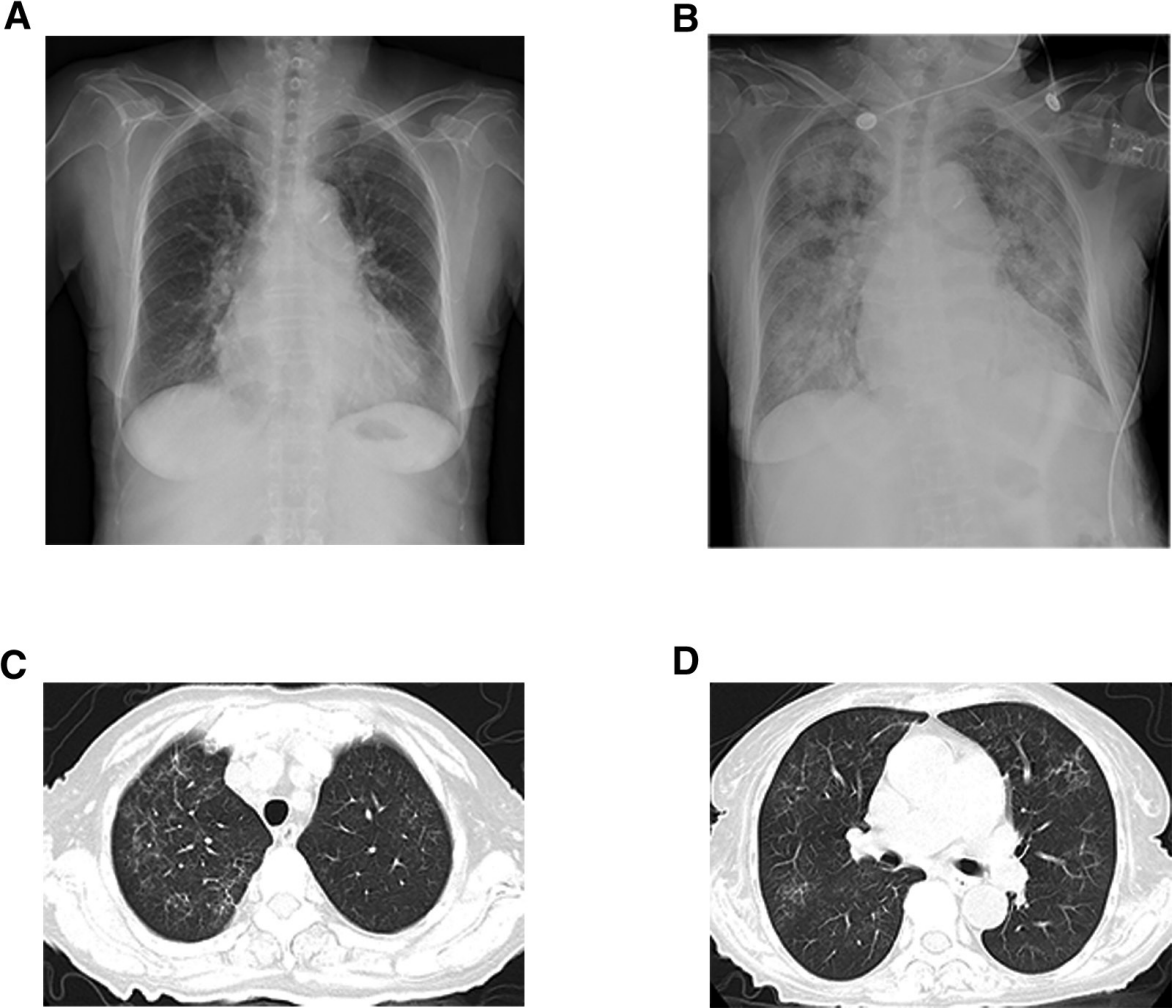
Radiographic and CT scan findings of the patient. (A) X-ray on July 13, 2023. (B) X-ray on July 27, 2023. (C) and (D) Chest CT scan results showing interstitial pneumonia on August 9, 2023.

Laboratory tests revealed a white blood cell count of 6.94 × 10^9^/L, hemoglobin count of 87 g/L, and platelet count of 194 × 10^9^/L. Biochemistry results showed potassium at 5.27 mmol/L, albumin at 30.9 g/L, globulin at 37.3 g/L; blood urea was 63.60 mmol/L, creatinine was 1856.00 μmol/L, C-reactive protein at 43.51 mg/L, and B-type natriuretic peptide precursor at 6670.0 ng/L. Chest radiography revealed a pear-shaped enlargement of the heart shadow and aortic sclerosis. Echocardiography revealed enlargement of the left atrium and a slightly thicker left ventricular wall, consistent with the ultrasonic changes in hypertensive heart disease. Abdominal ultrasonography revealed bilateral renal atrophy, with no abnormalities in the liver or spleen. Upon admission, the patient underwent hemodialysis. The hemodialysis program included the following components: a hollow fiber dialyzer (polyamide membrane, membrane area of 1.4 m²) with enoxaparin injection. After treatment, the patient’s edema decreased and fatigue and anorexia improved. However, she developed shoulder and knee joint pain, fever, and dense needle petechiae in both the lower limbs. By the eighth day of hospitalization, the platelet count had dropped to 80 × 10^9^/L. The platelet counts soon decreased to 8 × 10^9^/L, and C-reactive protein and procalcitonin levels increased significantly. Chest radiography revealed pulmonary edema and pulmonary parenchymal exudation, along with a significantly enlarged heart (Fig. 2). After the diagnosis of severe pneumonia with acute heart failure and secondary thrombocytopenia, the patient was transferred to the intensive care unit and treated with continuous renal replacement therapy without heparin. Imipenem was used as the anti-infection agent.

The patient’s platelet count continued to decline, reaching 0 on the 17th day of hospitalization. No significant increase was observed following platelet transfusions. Further examination revealed a positive heparin-induced thrombocytopenia (HIT)-related IgG antibody with an optical density of 0.70. Subsequently, the following data were obtained: antinuclear antibody (ANA), 1:640 with a fine speckled fluorescence pattern; anti-nucleosome antibody, 41.65 U/mL; anticardiolipin antibody, >300.00 AU/mL; anti-β2 glycoprotein I antibody, >300.00 AU/mL. Complement C3 was 0.405 g/L, and complement C4 was 0.048 g/L, both of which were significantly lower than the normal values. The erythrocyte sedimentation rate was > 140 mm/h. The Coombs test results showed a direct Coombs test (++), an indirect Coombs test (+), and positive platelet-specific and tissue-associated HLA antibodies (+). Anti-ds-DNA antibody, lupus anticoagulant, ANCA, lactic dehydrogenase, and anti-glomerular basement membrane antibodies tests were negative. The von Willebrand factor lyase activity (ADAMTS13) was 49.68%, and ADAMTS13 inhibitor was negative. Next-generation metagenomics sequencing of the blood revealed a cytomegalovirus ds-DNA sequence 342. The sputum culture identified Pseudomonas maltophilia. Blood cultures and tests for influenza A/B virus antigens, 2019 novel coronavirus nucleic acid, and dengue virus NS1 antigen were negative.

**Figure 3.**
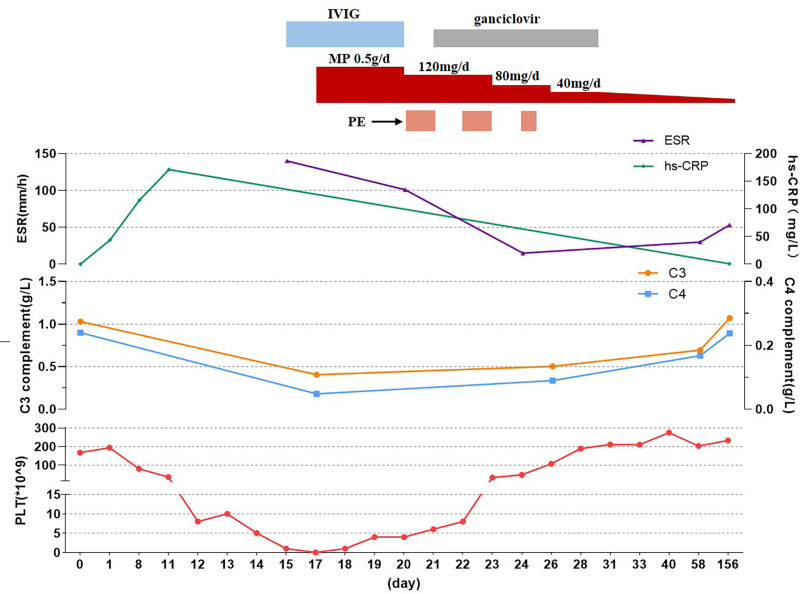
Clinical course during the treatment.

The patient was diagnosed with SLE complicated by hematological damage and immune thrombocytopenia. The 2019 EULAR/ACR classification criteria for SLE were met with a total score of 23 points, confirming the diagnosis of SLE. The treatment regimen was modified to include an intravenous infusion of 5% human immunoglobulin at 20 g/day for 5 days combined with an intravenous infusion of methylprednisolone at 500 mg/day for 3 days. The dose was then gradually reduced. Plasma exchange therapy was initiated on the 20th day of admission, conducted a total of 3 times, and ganciclovir capsules at 0.5 g every other day were administered to treat cytomegalovirus viremia. On the 23rd day, the patient’s platelet count began to increase, and by the 26th day, it returned to normal levels. Chest radiography showed significant absorption and reduction of exudative lesions in both lungs. On day 28, the patient was discharged on methylprednisolone (40 mg/day) and hydroxychloroquine (0.1 g bid). Low-molecular-weight heparin (LMWH) was continued, and the dose of glucocorticoids was gradually reduced. The platelet count was stable, ANA, anti-nucleosome antibody, antiphospholipid antibody, Coombs test, complement C3 and C4 were normal, and cytomegalovirus DNA was negative (Fig. 3).

## 3. Discussion

Thrombocytopenia can result from various conditions including drug-induced thrombocytopenia (e.g., HIT and sulfonamides), infections (such as HIV and hepatitis C), rheumatic diseases (including SLE and rheumatoid arthritis), and malignancies.

Diagnosing SLE in this case is challenging for several reasons: (1) the patient had normal ANA and complement levels in previous tests, a renal biopsy indicated IgA nephropathy, and there were no active clinical manifestations of SLE before this admission; (2) LMWH was used for anticoagulation during dialysis, and the patient’s platelets gradually decreased after the start of dialysis: HIT could not be excluded; and (3) the patient exhibited symptoms of infection, and some autoantibodies found in SLE can yield false positives under infectious conditions.

Before being diagnosed, LMWH was discontinued, continuous renal replacement therapy was initiated, and anti-infection treatment was administered. Shortness of breath improved, but the platelet count continued to decrease. The 4T score was 3, and the optical density of HIT-related IgG antibodies was 0.7, which did not support the diagnosis of HIT. Patients with acute parvovirus B19V (PVB19) infection can exhibit clinical signs and laboratory results similar to those seen in lupus.^[5]^ Although PVB19-specific IgM and IgG antibodies were not tested, metagenomic sequencing of the patient’s blood found no PVB19 sequences. Furthermore, the rapid platelet recovery following immunomodulatory therapies, such as corticosteroids and plasma exchange, strongly supports an autoimmune etiology over viral infection. These findings suggest that the decrease in platelet count cannot be solely attributed to infection or heparin induction. The patient did not exhibit any diarrhea or psychiatric symptoms. ADAMTS13 activity was 49.68%, and the ADAMTS13 inhibitor and lactic dehydrogenase levels were negative. Therefore, thrombotic microangiopathy was excluded from this study. The patient had not recently used sulfonamides, quinolones, vancomycin, or other drugs; therefore, there was no significant evidence of drug-induced thrombocytopenia.

The patient’s immunologic domains are unique in the following ways. First, this patient displayed a fine speckled ANA fluorescence pattern, commonly seen in various systemic autoimmune rheumatic diseases, in particular Sjögren’s syndrome, SLE, subacute cutaneous lupus erythematosus, and neonatal lupus erythematosus.^[6]^ However, antibodies of this patient such as anti-SSA/Ro and anti-SSB/La were negative, and the patient’s clinical and serological profile – including anti-nucleosome antibody positivity, hypocomplementemia, and thrombocytopenia – aligns decisively with SLE rather than Sjögren’s syndrome or other autoimmune rheumatic diseases.^[7]^ In addition, this patient tested negative for anti-ds-DNA antibodies, but their absence, though unusual, does not rule out SLE. Anti-nucleosome antibodies are a specific marker for SLE. Their presence in this patient, along with hypocomplementemia and antiphospholipid antibodies, provides strong support for the diagnosis. Furthermore, the frequency of anti-ds-DNA antibody negativity is elevated among elderly SLE patients, ranging from 30% to 40%.^[2]^

The incidence and cause of new-onset SLE during maintenance dialysis remain unclear. Generally, SLE activity decreases after dialysis initiation. Although the precise cause remains unclear, it may be related to reduced immune function in hemodialysis patients. Azotemia during dialysis suppresses immune responses.^[8]^ The immune system of hemodialysis patients is also weakened by functional suppression of granulocytes, macrophages, antigen-presenting cells, and B lymphocytes.^[9]^ However, new-onset SLE after the start of dialysis has been emerged^[10–19]^ (Table 1). Currently, there have been only 11 reported cases of new-onset lupus in hemodialysis patients, with the primary symptoms being fever and pericardial effusion. 6 patients, this 1 included, experienced thrombocytopenia >50%.^[10,13,17–19]^ Most patients with new-onset lupus have a favorable prognosis. Most patients showed clinical improvement following steroid therapy. However, 1 patient died of septic shock due to bacterial proctitis.^[18]^ This case represents the first documented successful treatment of thrombocytopenia induced by dialysis-onset lupus utilizing a combination of corticosteroids and plasma exchange. Further accumulation of cases is required to address this issue.

**Table 1:** Clinical characteristics, treatment and prognosis of 11 patients with new-onset SLE on hemodialysis.

Patient/Sex/Age, yrs	Duration of RRT, yrs	Primary disease	Disease manifestations	Immunoserology	Initial CS dose	IS therapy	Other treatment	Prognosis	Sources
0/F/69	0	IgA nephropathy	Co(fever), S(rash, stomatitis), M(arthritis), H(thrombocytopenia, hemolytic anemia)	ANA1:640, anti Anua 41.65 U/ml, hypocomplementemia, anticardiolipin antibody+, anti-β2GP1 antibody+	Methylprednisolone 0.5 g/d	NA	IVIG + PE	clinical improvement	Present case
1/F/18	2	Uncertainty	C(pericardial effusion), N(seizure, cerebrovascular disease), H(thrombocytopenia, leukopenia,)	ANA+, anti-DNA 1:2560	Prednisone, 1.5 mg/kg/d	NA	NA	clinical improvement, and renal transplantation	Julio el./1992
2/F/40	1	MPGN	Co(fever, general fatigue), S(hypersensitivity to sunlight, stomatitis), P(pleural effusion), C(pericardial effusion), H(leukopenia,)	ANA 1:1280, anti-DNA+, anti-sm+, anti-RNP+, hypocomplementemia	Prednisone	NA	NA	not shown	HIROSHI el./1993
3/M/36	4	MPGN	Co(fever), P(pleural effusion), H(hemolytic anemia), S(rash, Raynaud phenomenon)	ANA1:2560, positive anti-DNA, RF 1:640, hypocomplementemia, anticardiolipin antibody IgM	High dose (not presented)	CTX	NA	clinical improvement, died 1 year later with refractory arrhythmia	Richard el./1996
4/F/41	6	Uncertainty	Co(fever), M(arthritis), H(anemia, leukopenia)	ANA1:320, anti-dsDNA 283 IU/ml	Prednisone, 20 mg/d	MTX	NA	clinical improvement	David el./1997
5/M/38	14	CGN	Co(fever, generalized body aches), M(arthritis), H(thrombocytopenia, anemia, leukopenia)	ANA+, anti-DNA>100 μ/L, hypocomplementemia	High dose (not presented)	CTX for 6ws, azathioprine	NA	clinical improvement, died 2 years later with bleeding and sepsis	F. AI-Hawas el./1997
6/M/70	4	DKD	Co(fever), M(arthritis), P(pleural effusion), C(pericardial effusion)	ANA+, anti-dsDNA 45 IU/ml	Prednisone, 40 mg/d	NA	NA	clinical improvement	Maki el./2013
7/F/61	25	Uncertainty	Co(fever), M(arthritis), P(pleuritis, pneumonitis), H(anemia, leukopenia)	ANA1:2560, anti-dsDNA 59.7 U/ml,	Methylprednisolone 1 g/d	NA	NA	clinical improvement	Hiroto el./2015
8/F/71	2	PKD	Co(fever, fatigue), H(thrombocytopenia, anemia, leukopenia), P(pleural effusion), C(pericardial effusion), S(nodules)	ANA>1:640	Prednisone, 35 mg/d	NA	NA	clinical improvement	Eleni el./2015
9/F/61	4	Uncertainty	Co(fever), P(pleural effusion), C(pericardial effusion), G(pericardial effusion), H(thrombocytopenia)	ANA1:2560, anti-dsDNA 354i U/ml	Prednisone, 50 mg/d	NA	meropenem	level of anti dsDNA decreased, but died by bacterial proctitis-induced septic shock shortly thereafter	Shotaro el./2021
10/F/35	2	CGN	Co(fever), S(spontaneous ecchymosis), H(thrombocytopenia, leukopenia, hemolytic anemia, psoas muscle hematoma), P(pleural effusion), C(pericardial effusion)	ANA+,anti-DNA+, hypocomplementemiaC3	Methylprednisolone	MMF 1 g/d	NA	clinical improvement	Marouane el./2023

C = cardiac, CGN = chronic glomerulonephritis, Co = constitutional, CS = corticosteroid, CTX = cyclophosphamide, DKD = diabetic kidney disease, H = hematologic, IS = immunosuppressive, IVIG = intravenous immunoglobulin, M = musculoskeletal, MMF = mycophenolate mofetil, MPGN = membranoproliferative glomerulonephritis, NA = not applicable, P = pulmonary, PE = plasma exchange, PKD = polycystic kidney disease, RRT = renal replacement therapy, S = skin, SLE = systemic lupus erythematosus.

In conclusion, we describe the case of an elderly woman with end-stage renal failure who developed new-onset SLE after starting hemodialysis. In patients with unexplained thrombocytopenia, the possibility of SLE should be considered, even after the initiation of hemodialysis and after ruling out possible factors such as infection, HIT, and thrombotic microangiopathy.

## Acknowledgments

We would like to express our gratitude to the patient for granting permission to use their clinical data in this paper and for the publication.

## Author contributions

**Data curation:** Zhe Zhang, Peiyi Ye.

**Resources:** Yunshi Lai, Xiaoyi Liu.

**Software:** Zhe Zhang, Xiaoyi Liu.

**Supervision:** Yaozhong Kong, Chao Xie.

**Writing – original draft:** Zhe Zhang, Yunshi Lai.

**Writing – review & editing:** Chao Xie.
